# Chitosan Microsphere Used as an Effective System to Deliver a Linked Antigenic Peptides Vaccine Protect Mice Against Acute and Chronic *Toxoplasmosis*

**DOI:** 10.3389/fcimb.2018.00163

**Published:** 2018-05-23

**Authors:** Jingjing Guo, Xiahui Sun, Huiquan Yin, Ting Wang, Yan Li, Chunxue Zhou, Huaiyu Zhou, Shenyi He, Hua Cong

**Affiliations:** Department of Human Parasitology, Shandong University, School of Medicine, Jinan, China

**Keywords:** *Toxoplasma gondii*, chitosan microspheres, peptides, epitopes, vaccine

## Abstract

Multiple antigenic peptide (MAP) vaccines have advantages over traditional *Toxoplasma gondii* vaccines, but are more susceptible to enzymatic degradation. As an effective delivery system, chitosan microspheres (CS) can overcome this obstacle and act as a natural adjuvant to promote T helper 1 (Th1) cellular immune responses. In this study, we use chitosan microparticles to deliver multiple antigenic epitopes from GRA10 (G10E), containing three dominant epitopes. When G10E was entrapped within chitosan microparticles (G10E-CS), adequate peptides for eliciting immune response were loaded in the microsphere core and this complex released G10E peptides stably. The efficiency of G10E-CS was detected both *in vitro*, via cell culture, and through *in vivo* mouse immunization. *In vitro*, G10E-CS activated Dendritic Cells (DC) and T lymphocytes by upregulating the secretion of costimulatory molecules (CD40 and CD86). *In vivo*, Th1 biased cellular and humoral immune responses were activated in mice vaccinated with G10E-CS, accompanied by significantly increased production of IFN-γ, IL-2, and IgG, and decreases in IL-4, IL-10, and IgG1. Immunization with G10E-CS conferred significant protection with prolonged survival in mice model of acute toxoplasmosis and statistically significant decreases in cyst burden in murine chronic toxoplasmosis. The results from this study indicate that chitosan microspheres used as an effective system to deliver a linked antigenic peptides is a promising strategy for the development of efficient vaccine against *T. gondii*.

## Introduction

*Toxoplasma gondii* is an important medical pathogen that infects approximately 30% of the global population. Generally, toxoplasmosis is asymptomatic in immune-competent hosts, however, it can result in severe symptoms in immunocompromised individuals due to cerebral cyst reactivation. Another potentially fatal presentation is vertical transmission in the fetus, which can result in encephalitis, neonatal malformations, or spontaneous abortion (Blader et al., 2015; Dimier-Poisson et al., 2015). Current drug treatment cannot control this disease completely because of the inability of drugs to kill bradyzoites (Henriquez et al., 2010). Therefore, the advantages of a preemptive vaccine for preventing toxoplasmosis are obvious (Innes, 2010). Traditional vaccine development strategies against *T. gondii* mainly focused on subunit and DNA vaccines. Their use raises several issues, since subunit vaccines have poor stability and may cause undesired immune responses (Skwarczynski and Toth, 2014), and DNA vaccines have the theoretical risk of genomic integration into host cells (Kofler et al., 2004).

Peptide-based vaccines could overcome these weaknesses. They use minimal antigenic epitopes to induce desired immune responses, and are less likely to trigger allergic or autoimmune responses (Skwarczynski and Toth, 2016). In recent years, there has been increasing interest in the study of peptide-based vaccines (Dudek et al., 2010). *T. gondii* is an intracellular parasite with a complex life cycle, so synthetic multiple antigenic peptide (MAP) vaccines containing different epitopes may prove a highly efficacious strategy in the development of *T. gondii* vaccines (Henriquez et al., 2010).

Successful *T. gondii* infection requires active invasion and the formation of the parasitophorous vacuole (PV) (Braun et al., 2008). Dense granules are secretory organelles involved in maturation and modification of both the PV and its membrane (PVM) (Nam, 2009). Dense granules proteins (GRAs) are major components of the vacuole surrounding tachyzoites and encysted bradyzoites, and are related to host-parasite interactions and immune responses (Cesbron-Delauw and Capron, 1993). Dense granule protein 10 (GRA10) which is released into the PV shortly after invasion and then localizes to the PVM (Ahn et al., 2005), is essential for parasite growth with potential immunogenic capability. Researchers found that there is severe inhibition of *T. gondii* growth in human fibroblasts cells when GRA10 is knocked out (Witola et al., 2014). Of note, immunogenic peptides from GRA10 in a vaccine formulation have not been previously explored.

However, peptides are very susceptible to enzymatic degradation. Thus, a delivery system is needed to protect protease-sensitive epitopes from degradation (van Riet et al., 2014; Skwarczynski and Toth, 2016). Recently, microparticles as a delivery system to load antigens has emerged as one of the most promising strategies to induce a strong immune response (Kwon et al., 2005; Reddy et al., 2007). In this way, the peptides are delivered by microspheres, thereby inducing enhanced recognition by the immune system compared with naked easy degradation peptides (van der Lubben et al., 2001). Thus far, many types of microparticles have been tested, and chitosan has attracted particular interest. Chitosan is the deacetylated form of chitin, a naturally occurring and abundantly available biocompatible polysaccharide (Shrestha et al., 2014). Chitosan microspheres have many advantages over traditional vaccine microsphere formulations with starch, gelatin, or albumin. They are easy to load with peptides, thereby circumventing protein denaturation (Lin et al., 2013). Compared to other biodegradable polymers, chitosan is the only one that has a cationic (Bernkop-Schnürch and Dünnhaupt, 2012) and mucoadhesive character, increasing residual time at the site of absorption, therefore prolonging the release time of protein antigens (Agnihotri et al., 2004). When chitosan degrades, the amino sugars produced are nontoxic and easily removable from the body without causing side reactions (Wang et al., 2016). More importantly, chitosan is a demonstrated natural adjuvant to promote dendritic cell (DC) activation and T helper 1 (Th1) cellular-associated immune responses via cGAS-STING signaling pathway (Carroll et al., 2016; Riteau and Sher, 2016). Collectively, as an attractive carrier and adjuvant, chitosan has been used extensively in vaccine applications (Islam and Ferro, 2016).

Herein, we present a novel attempt to combine highly immunogenic MAP from GRA10 with chitosan microspheres in the design of a vaccine against *T. gondii*. In this study, three dominant epitopes from GRA10 were screened from nine potential epitopes and were used to synthesize the MAP of GRA10 (G10E). The release efficiency of these chitosan microparticles containing the MAP of GRA10 (G10E-CS) was detected in terms of peptide loading and releasing efficiency. As an adjuvant, the capacity of chitosan to activate dendritic cells and T lymphocytes was tested *in vitro*. Furthermore, the protective effect of G10E-CS vaccination on acute and chronic toxoplasmosis murine models was evaluated. This study aims to make a contribution to peptides vaccines against *T. gondii* by exploring chitosan microspheres, an effective delivery system, to enhance MAP vaccine immunogenicity.

## Materials and methods

### Animals

Female 6–8weeks-old BALB/c and C57BL/6 mice were purchased from Shandong University Laboratory Animal Centre (Jinan, China) and housed in SPF conditions. All the mice were housed 5 per cage under pathogen-free conditions and were adequately supplied with sterilized water and food.

### Ethical approval

Experiments were carried out in accordance with animal ethics approved by the Institutional Animal Care and Use Committee of Shandong University under Contract LL201602044. Humane endpoints were chosen to terminate the pain or distress of experimental animals via euthanasia.

### Parasite

Tachyzoites of the high-virulence RH strain (type I) (Howe and Sibley, 1995) of *T. gondii* were propagated in human foreskin fibroblast (HFF) cells in Dulbecco's modified Eagle's medium (DMEM) (Sigma-Aldrich, USA) supplemented with 10% fetal bovine serum (FBS) (Clark Biosciences,USA) as previously described (Shen and Sibley, 2014). Pru (Prugniaud) strain (type II), a low-virulence strain of *T. gondii*, (Saeij et al., 2005)was a kind gift from Professor Xingquan Zhu at Lanzhou Veterinary Research Institute, China. The cysts of the type II Pru strain were maintained in Kunming mice by oral passage of infectious cysts in mice as previously described (Zhou et al., 2016).

### Screening of candidate epitopes from GRA10

The PROTEAN subroutine in the DNASTAR software package was used to predict the B cell epitopes of GRA10 (TGME49_268900, ToxoDB) as described previously (Wang et al., 2014). Three B cell candidate epitopes, GRA10_192−215_ (P1), GRA10_221−244_ (P2), and GRA10_310−332_ (P3), were selected with good hydrophilicity, high conformational flexibility, surface accessibility and strong antigenicity.

To predict the CD8^+^ and CD4^+^ T cell epitopes of GRA10, the complete amino acid sequence of the protein was analyzed using consensus algorithms available at the Immune Epitope Database and Analysis Resource (IEDB, http://www.iedb.org/) as described previously (Cong et al., 2010). GRA10_49−57_ (P4), GRA10_161−169_ (P5), and GRA10_300−308_ (P6) were chosen based on their predicted binding affinity to MHC I supertype molecules (those with a percentile rank lower than 10 were selected; Moutaftsi et al., 2006). GRA10_80−94_(P7), GRA10_170−184_(P8), and GRA10_180−194_(P9) were chosen based on their predicted binding affinity to MHC II (Wang et al., 2008). The peptides (P1-P9) sequences are shown in Table 1 and were synthesized (>95% purity) by Synpeptide Co., Ltd. (Shanghai, China) and stored at −80°C.

**Table 1:** Candidates of B cell or T cell epitopes predicted from GRA10 of *T. gondii*.

**Type**	**Epitope**	**Sequence**
B cell epitope^a^	GRA10_192−215_	TQSPPESRKKRRRSGKKKRGKRSV(P1)
	GRA10_221−244_	GSGTLPSDEPVDGCDRVREEAERE(P2)
	GRA10_310−332_	GDKNDTDTTQNKDTGSTQSQRAN(P3)
CD8^+^ T cell epitope^b^	GRA10_49−57_	LPKKGVPVG(P4)
	GRA10_161−169_	LGYCALLPL(P5)
	GRA10_300−308_	VPPVLKISR(P6)
CD4^+^ T cell epitope^c^	GRA10_80−94_	SGFSLSSGSGVSVVE(P7)
	GRA10_170−184_	LTEEQFRHIRRLQKR(P8)
	GRA10_180−194_	RLQKRAMVLCGYTQS(P9)

a *Three B cell candidate epitopes, GRA10_192-215_ (P1), GRA10_221-244_ (P2), and GRA10_310-332_ (P3 with good hydrophilicity, high conformational flexibility, surface accessibility and strong antigenicity, were selected via DNASTAR software*.

b,c *CD8^+^ and CD4^+^ T cell epitopes of GRA10 were analyzed using consensus algorithms available at the Immune Epitope Database and Analysis Resource (IEDB, http://www.iedb.org/)*.

ELISA analysis of serum samples from *T. gondii* infected mice was used to screen three GRA10 B cell epitopes as previously described (Cardona et al., 2009). IgG OD value was recorded as the absorbance at 450 nm detected by a Thermo Scientific Multiskan FC Microplate Photometer (Thermo Scientific, USA). Meanwhile, lymphocyte proliferation assays of infected mice were used to screen GRA10 CD8^+^ T cell epitopes and CD4^+^ T cell epitopes as previously described (Wallace et al., 2008).

### Construction of multiple antigenic peptide (MAP) of GRA10 (G10E)

**Figure 1:**
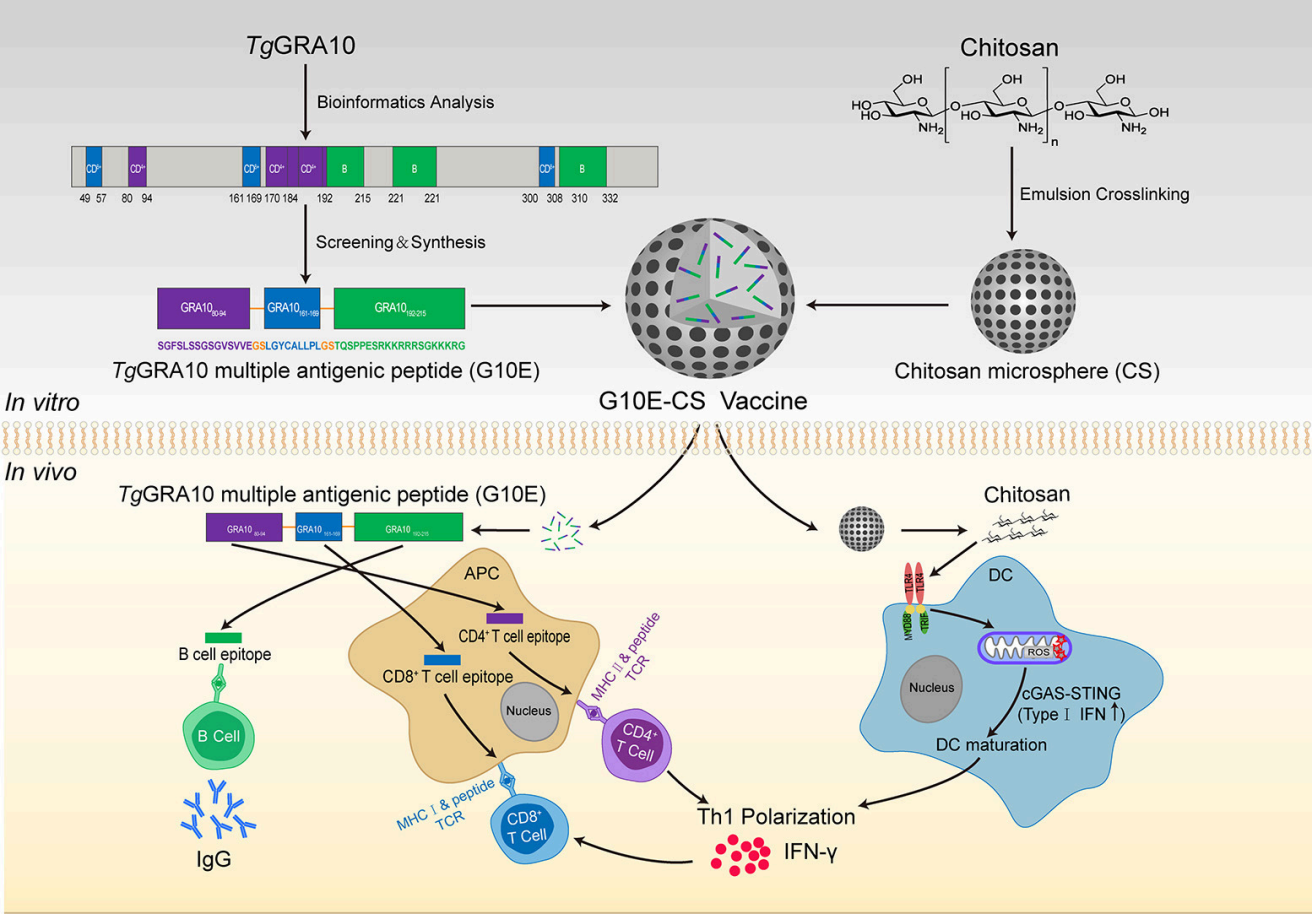
Construction of *T. gondii* G10E-CS vaccine and potential immune mechanism involved in vaccination with G10E-CS *in vivo*. In this study, dominant B cell, CD8^+^ and CD4^+^ T cell epitopes from GRA10 protein of *T. gondii* were selected by bioinformatics and immunological analysis. Then the G10E-CS microsphere vaccine formed by three dominant epitopes and linked by sequence GS was loaded onto chitosan microspheres by emulsion cross-linking method. When BALB/c mice were immunized with G10E-CS, the G10E peptides released from the microspheres induced specific humoral and cellular response. At the same time, chitosan as an attractive adjuvant could induce activation of dendritic cells (DCs) via cGAS-STING signaling pathway and then promote T helper 1 (Th1) cellular immune responses.

*T. gondii* GRA10 MAP (G10E) was designed by linking the selected epitopes with the spacer sequence Gly-Ser (Figure 1) and then synthesized by Synpeptide Co., Ltd. (Shanghai, China) and stored at −80°C.

### Preparation of chitosan microspheres loaded with G10E (G10E-CS)

Chitosan microspheres loaded with G10E were prepared by emulsion crosslinking method as previously described (Jose et al., 2013; Pichayakorn and Boonme, 2013) with minor modifications. Briefly, chitosan solution (3% w/w) was prepared by dissolving chitosan in 2% aqueous glacial acetic acid as aqueous phase. G10E peptides were mixed in this solution. Oil phase was paraffin oil containing 5% Span80. The aqueous phase was gradually added into the oil phase and continuously mixed at 1,000 rpm for 2h to form water-in-oil (w/o) emulsion. Subsequently, genipin was added into the mixture and mixed further for 2 h. The obtained microspheres were then separated by centrifugation at 3,000 rpm and the sediment was washed thrice with petroleum ether, isopropanol and deionized water and finally dried using a freeze dryer (CHRIST ALPHA 1-4 LSC, Germany) (Figure 1).

G10E-CS microspheres were characterized for morphological shape by a scanning electron microscope (JSM6610LV SEM, JEOL Ltd., Japan) as previously described (Wang et al., 2005). The samples were dusted on an adhesive carbon tape and coated with a thin layer of gold. Samples were then imaged at magnification of 15,000. The particle size and zeta potential of chitosan microspheres was determined by dynamic light scattering on a Zetasizer NanoZS (Malvern, UK). Measurements were carried out in triplicate.

### Loading capacity and encapsulation efficiency

1–5 mg/ml G10E peptides were mixed with 3% (w/v) chitosan microspheres and crosslinked by genipin as above described. The suspension was centrifuged (3,000 rpm for 30 min) to collect the supernatant. Then, the non-bound G10E concentration in the supernatant was determined by Protein Quantitative Reagent Kit-BCA Method (Beyotime, China) (Wang et al., 2016). Loading capacity (LC) and encapsulation efficiency (EE) in chitosan microspheres were determined by applying the following equations:

$$\begin{array}{l} \text{Loading capacity }\left(\%\text{LC}\right)=\\ \frac{\left(\text{total amount G1}0\text{E}\right)-\left(\text{free G1}0\text{E}\right)}{\text{weight of microparticles}}\;\times \;100\%\\ \text{Encapsulation efficiency }\left(\%\text{EE}\right)=\\ \frac{\left(\text{total amount G1}0\text{E}\right)-\left(\text{free G1}0\text{E}\right)}{\text{total G1}0\text{E}}\;\times \;100\% \end{array}$$

### Evaluation of release characteristics of G10E *in vitro*

G10E release from chitosan microparticles *in vitro* was determined in PBS (pH 7.4) as previously described (Skop et al., 2013) with minor modifications. Genipin-crosslinked G10E-CS microspheres and G10E peptides were dispersed in PBS (pH 7.4) at 37°C under mild shaking (100 rpm). At specific time intervals, 250 μl of the supernatant containing the released G10E was taken by centrifugation and stored at −20°C in tubes. The chitosan microspheres that settled at the bottom of the tube were resuspended in PBS (pH 7.4) after each collection. After the last time point, the protein concentration of all the collected G10E was analyzed using the Protein Quantitative Reagent Kit-BCA Method.

The degradation of released G10E was analyzed by gel electrophoresis. Briefly, G10E-CS microspheres were dispersed in PBS (pH 7.4) using a shaking air bath (37°C, 100 rpm) for 15 d. After centrifuging (5,000 rpm, 20 min), the microsphere precipitate at the bottom of the tube was collected to be analyzed by gel electrophoresis.

### The expression of costimulatory molecules on the surface of stimulated dendritic cells

Bone-marrow derived dendritic cells (DCs) were prepared as previously described (Molavi et al., 2010). Briefly, C57BL/6 mice were euthanized, intact femurs were removed, and purified of surrounding muscle tissue. The bones were washed with sterile PBS, the bone marrow cells were harvested by repeated flushing with RPMI 1640 using a syringe with a 0.45 mm diameter needle. To derive conventional DCs, bone marrow cells (2 × 10^6^ cells/ml) were cultured for 7 days in 6-well plate (Corning Inc., USA) in RPMI 1640 medium (Hyclone, USA) supplemented with 10% FBS (Hyclone, USA), 50 μM penicillin and streptomycin, and 20 ng/ml GM-CSF and IL-4 (Sigma-Aldrich, USA). Half of the medium was replaced with fresh complete medium on day 3 and 5. At day 7, non-adherent cells were harvested and ready for use as mature DCs.

To analyse costimulatory molecule expression, dendritic cells (2 × 10^6^ cells/ml) were seeded in 96-well plate (Corning Inc., USA) in the same medium as described above (including GM-CSF and IL-4) and cultured with CpG (4 μg/mL), CS, G10E or G10E-CS (10 μg/mL each) for 48 h. CpG, a TLR agonist that generally enhances CD40 and CD86 surface expression, served as a positive control. Cell suspension was subsequently stained with 0.3 μg/ml of anti-CD11c, 1.25 μg/ml of anti-CD86 and 2.5 μg/ml of anti-CD40 antibody (BD Biosciences, USA) (Carroll et al., 2016). Samples were detected by Cytoflex S Flow Cytometer (Beckman Coulter, USA) and the data were analyzed by CytExpert software.

### Activation of dendritic cells and lymphocyte *in vitro* induced by G10E-CS microspheres

Dendritic cells (DCs) were plated at 2 × 10^5^ cells per well and stimulated as described above. At the same time, lymphocytes isolated from the spleen of C57BL/6 mice were plated in 96-well plates, at a density of 2 × 10^6^ cells/ml, in 100 μL RPMI-1640 medium supplemented with 10% FBS, 50 μM penicillin and streptomycin. Then, induced DCs were cocultured with these T cells (1:10 ratio) for 72 h (Höpken et al., 2005), and lymphocyte proliferation was measured by CCK-8 assay. In the meantime, supernatants of mixed cells were assayed for IL-2 and IFN-γ using a commercial ELISA Kit (R&D Systems, USA) following the procedure recommended by the manufacturer.

### Cytotoxicity of G10E-CS microspheres

The cytotoxicity of the G10E-CS microspheres *in vitro* was evaluated by CCK-8 assay (Dojindo, Japan) conducted on the aforementioned dendritic cells (DCs) and T lymphocytes as previously described (Shrestha et al., 2014). Briefly, these cells were added to 96-well plates of 2 × 10^6^ cells/ml at 100 μL per well and incubated overnight. The culture medium was then replaced by serum-free medium with different concentrations (125, 250 μg/mL and 500 μg/mL) of G10E-CS microspheres and incubated for 48 and 72 h at 37°C. Ten micro liters CCK-8 was added to the wells and the absorbance of the solution was measured at 450 nm in a Thermo Scientific Multiskan FC Microplate Photometer (Thermo Scientific, USA). Cell viability (%) was defined as the OD ratio in test group relative to controls. All experiments were carried out five times.

### Immunization and challenge infection in mice

BALB/c mice were randomly divided into four groups (*n* = 23 per group) and were immunized thrice via intramuscular injection at 2-week intervals with G10E (100 μg), G10E-CS microspheres containing 100 μg of G10E (667 μg) (LC = 15%) or empty CS microsphere (667 μg) (Zhou et al., 2007; Jose et al., 2012). Mice that received 100 μl PBS were used as non-immunized control.

Two weeks after the last immunization, the protection of G10E-CS microspheres against acute and chronic *T. gondii* infection was investigated as previously described (Wang J. L. et al., 2017). Ten mice per immunized group were infected intraperitoneally with a lethal dose of RH strain (1 × 10^3^ tachyzoites). The challenged mice were monitored over a period of 21 days and observed daily for mortality.

At the same time, 10 mice per group were challenged orally with a sublethal dose of cysts (30 cysts) of the PRU strain (type II) to evaluate the effect of vaccination in mice with chronic toxoplasmosis. All surviving mice were euthanized at 45 days after challenge. Brains were collected and homogenized in 1 ml PBS. The number of cysts per brain was confirmed by counting eight 10 μl samples of each cerebral homogenate under an optical microscope.

### Detection of total IgG and antibody isotypes in vaccinated mice

Serum samples were collected from the mice by retro-orbital bleeding at 0, 2, 4, and 6 weeks after immunization. Standard ELISA assay was used to determine the levels of *T. gondii*-specific IgG, IgG1 and IgG2a antibodies in the serum samples as previously described (Tang et al., 2016). Briefly, 96-well plates were coated with the 100 μL (10 μg/mL) G10E peptides in 50 mM carbonate-bicarbonate buffer (pH 9.6) and were incubated at 4°C overnight. After washing three times with PBS plus 0.05% Tween 20 (PBST), the coated plates were blocked with 1% low fat milk in PBST for 1 h at 37°C. The mouse serum was diluted in PBS (1:25) and incubated at 37°C for 1 h. Then plates were washed, and anti-mouse-IgG, IgG1 or IgG2a HRP-conjugated antibodies (Sigma-Aldrich, USA) were added to each well. Peroxidase activity was revealed by 10 mg/ml 3, 3′, 5, 5′-tetramethylbenzidine (TMB, Sigma–Aldrich, USA) and stopped by adding 50 μl of 2 M H_2_SO_4_. The results were recorded as the absorbance at 450 nm and detected by a Thermo Scientific Multiskan FC Microplate Photometer (Thermo Scientific, USA). For each serum sample the assay was done in triplicate and average values were calculated.

### Lymphocyte proliferation assay

Two weeks after the last immunization, three mice per group were euthanized to obtain single splenocyte suspensions as described previously (Luo et al., 2017). After erythrocytes were lysed, the splenocytes were adjusted to a concentration of 2 × 10^6^ cells/ml in RPMI-1640 medium with 10% FBS and plated in 96-well plates in presence of the G10E peptides (10 μg/mL). Plates were incubated for 72 h at 37 °C. Lymphocyte proliferative activity was measured by a Cell Counting Kit-8 (CCK-8, Dojindo; Japan) according to manufacturer's instructions. The absorbance was detected at 450 nm and the stimulation index (SI) was calculated.

### Flow cytometry analysis of T cell subsets and cytokine production

The percentages of CD4^+^ and CD8^+^ T lymphocytes subsets and the production of cytokine in the splenocytes of immunized mice were determined using flow cytometry techniques as described previously (Zhang et al., 2015b; Wang S. et al., 2017). Briefly, splenocytes (2 × 10^6^cells/ml) of vaccinated mice were stimulated with G10E peptides (10 μg/mL) for 72 h. Then the suspensions were stained with anti-mouse CD3-APC, anti- mouse CD4-FITC and anti-mouse CD8-PE (eBiosciences, USA) for 30 min at 4 °C in the dark.

Splenocytes (2 × 10^6^ cells/ml) were also stimulated with G10E peptides for 72 h in the presence of Cell Stimulation Cocktail (eBiosciences, USA) containing Phorbol myristate acetate (PMA, 20 ng/ml), Ionomycin (2 μg/ml), Brefeldin A (1 μg/ml), and Monensin (1 μg/ml) to inhibit the secretion of cytokine into the extracellular space. The cells were fixed using an Intracellular Fixation & Permeabilization Buffer Set Kit in accordance with the manufacturer's protocol (eBiosciences, USA) and then stained directly with anti-mouse CD4-FITC, anti-mouse CD8-PE, anti-mouse IL-2 (APC), anti-mouse IFN-γ (PerCP-Cyanine 5.5), anti-mouse IL-4 (APC), and anti-mouse IL-10 (PerCP-Cyanine5.5) (eBiosciences, USA) for 30 min at 4°C. All these cell population were analyzed by Cytoflex S Flow Cytometer (Beckman Coulter, USA) and the data were analyzed by CytExpert software.

### Statistical analysis

Statistical analysis was carried out using SPSS19.0 (SPSS Inc., Chicago, IL, USA) and GraphPad Prism 7.0 (GraphPad Software Inc., San Diego, USA). Differences between groups were analyzed using one-way ANOVA analysis. Student's *t*-test was used for comparison between levels of IgG1 and IgG2a isotypes. Survival percentage was analyzed by the Kaplan-Meier test and survival curves were compared using the Log-rank test. Data are expressed as means ± SD. Values of *p* < 0.05 were considered statistically significant.

## Results

### Epitopes screened from *Tg*GRA10 and construction of multiple antigenic peptide

**Figure 2:**
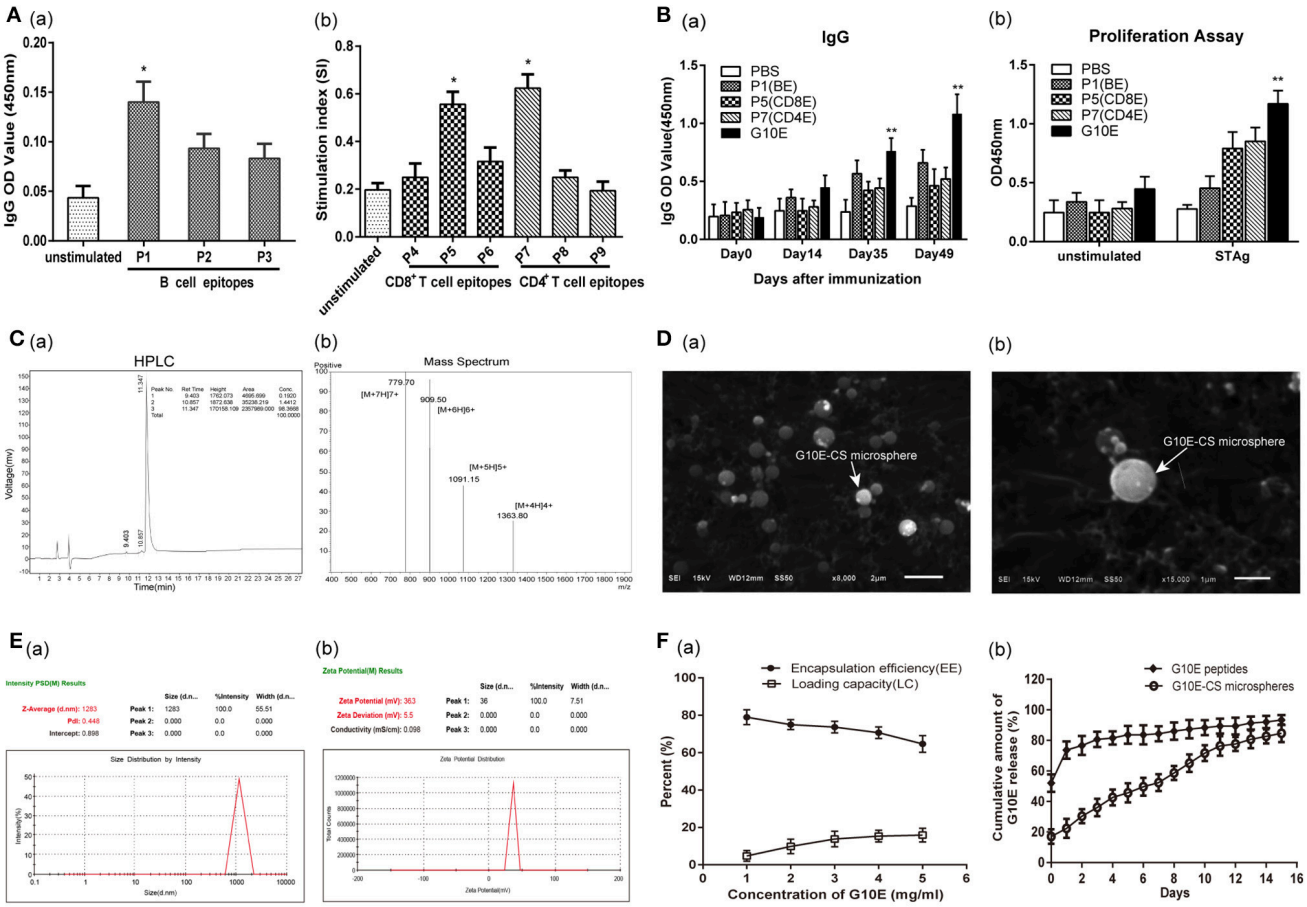
Screening of dominant epitopes from GRA10 and characteristic of G10E-CS. **(A)** Screening of the dominant B cell epitopes **(a)** and T cell epitopes **(b)** from TgGRA10. Positive serum samples from *T. gondii* infected mice were used to screen dominant B cell epitopes by ELISA analysis. Lymphocyte proliferation test was used to identify dominant CD8^+^ T and CD4^+^ T cell epitopes from spleen cells of infected mice by CCK-8 assay. P1(TQSPPESRKKRRRSGKKKRGKRSV)(B cell epitope), P5(LGYCALLPL) (CD8^+^ T cell epitope) and P7(SGFSLSSGSGVSVVE) (CD4^+^ T cell epitope) were selected to construct the multiple antigenic peptide (MAP) of *T. gondii* GRA10 (G10E). Values represent mean ±SD (*n* = 5). *p < 0.05. **(B)** Immunogenicity of P1, P5, P,7 and G10E peptides. Mice immunized with P1(B cell epitope), P5(CD4+ T cell epitope), P7(CD8+ T cell epitope) or G10E (P5-P7-P1) were tested for B cell antibody **(a)** and T cell proliferation ability **(b)**.**p < 0.01. **(C)** The purity of G10E peptides detected by high performance liquid chromatography (HPLC) **(a)** and the molecular weight of G10E peptides analyzed by electrospray ionization mass spectrometry (ESI-MS) **(b)**. **(D)** Scanning electron microscopy (SEM) images of G10E-CS microspheres prepared by emulsion crosslinking method. G10E-CS microspheres were then imaged at magnification of ×15,000 (bar represents 1 μm). **(E)** The diameter **(a)** and zeta of G10E-CS **(b)** microparticles was detected by Malvern particle size potentiometer. **(F)** Loading capacity (%LC) and encapsulation efficiency (%EE) of G10E-CS microspheres amongst 1–5 mg/ml concentration of total G10E peptides **(a)**. Release profile of G10E peptides from free G10E peptides and crosslinked chitosan microparticles (G10E-CS) *in vitro* over a 15-day period **(b)**. The amount of G10E in the supernatant was measured using BCA assay. Values represent mean ± SD (*n* = 3).

The peptide sequences of candidate epitopes from GRA10 showing high affinity in the prediction algorithm in Table 1 were further tested in mice. The IgG test showed that all the B cell epitopes from GRA10 could recognize the IgG in the serum of BALB/c mice infected by *T. gondii*, in which GRA10_192−215_ (P1) was demonstrated to induce highest level of IgG (Figure 2Aa). Lymphocyte proliferation testing showed that GRA10_161−169_ (P5) and GRA10_80−94_ (P7) stimulated the highest level of proliferation of lymphocytes in infected mice (Figure 2Ab).

Thus, GRA10_192−215_ (P1), GRA10_161−169_ (P5), and GRA10_80−94_ (P7) were chosen to construct the MAP G10E with linker GS (Figure 1). Mice immunized with P1(B cell epitope), P5(CD4^+^ T cell epitope), P7(CD8^+^ T cell epitope) or G10E were tested for B cell antibody level and T cell proliferation ability. The results showed that significant enhanced B cell and T cell immune responses were achieved in G10E vaccinated group (Figures 2Ba,b).

### Physical characterization and release of chitosan microspheres loaded with G10E

Chitosan microspheres loaded with G10E peptides (G10E-CS) were prepared by emulsion cross-linking method. The purity of G10E was 98.4% detected by high performance liquid chromatography (HPLC) (Figure 2Ca), and its molecular weight was 5450.9 daltons, analyzed by electrospray ionization mass spectrometry (ESI-MS) (Figure 2Cb). The morphology of G10E-CS microspheres formed using 3% chitosan was spherical in shape with a smooth surface, as observed by scanning electron microscopy (SEM) (Figure 2D). The average diameter of G10E-CS microparticles was 1283 nm with the low dpi detected by Malvern particle size potentiometer, suggesting that G10E-CS were uniform in size (Figure 2Ea).

The zeta potential of the CS microspheres was positive 37.1 ± 5.2 mV while the G10E peptides was negative 31.7 ± 9.1 mV (Table S1). After loading G10E into chitosan microspheres, the G10E-CS's zeta potential were positive 36.0 ± 5.48 mV (Figure 2Eb). Size and zeta analysis of G10E-CS formulations confirmed that G10E was completely loaded into the core of the CS microspheres, and not simply adherent to their surfaces.

The loading capacity and encapsulation efficiency of the chitosan microparticles were analyzed. When the concentration of G10E reached 4 mg/ml, a high encapsulation efficiency (EE) of 71% was achieved, and the peptide loading was almost saturated (15.3%). Therefore, 4 mg/ml G10E peptides were selected as the optimal concentration (Figure 2Fa).

We further evaluated the release of the entrapped G10E peptides from the chitosan microparticles. The crosslinked G10E-CS microspheres showed the more favorable and steady release profile (Figure 2Fb). Only 22.4% of the total encapsulated G10E peptides were released within the first day and slowly released 2.4–6.9% peptides daily over the next 14 days. While G10E peptides released 80% in the first 2 days in the freedom style This was further supported by PAGE and SDS-PAGE analysis of released G10E (Figure S1).

### G10E-CS microspheres induce the activation of dendritic cells and lymphocyte *in vitro*

**Figure 3:**
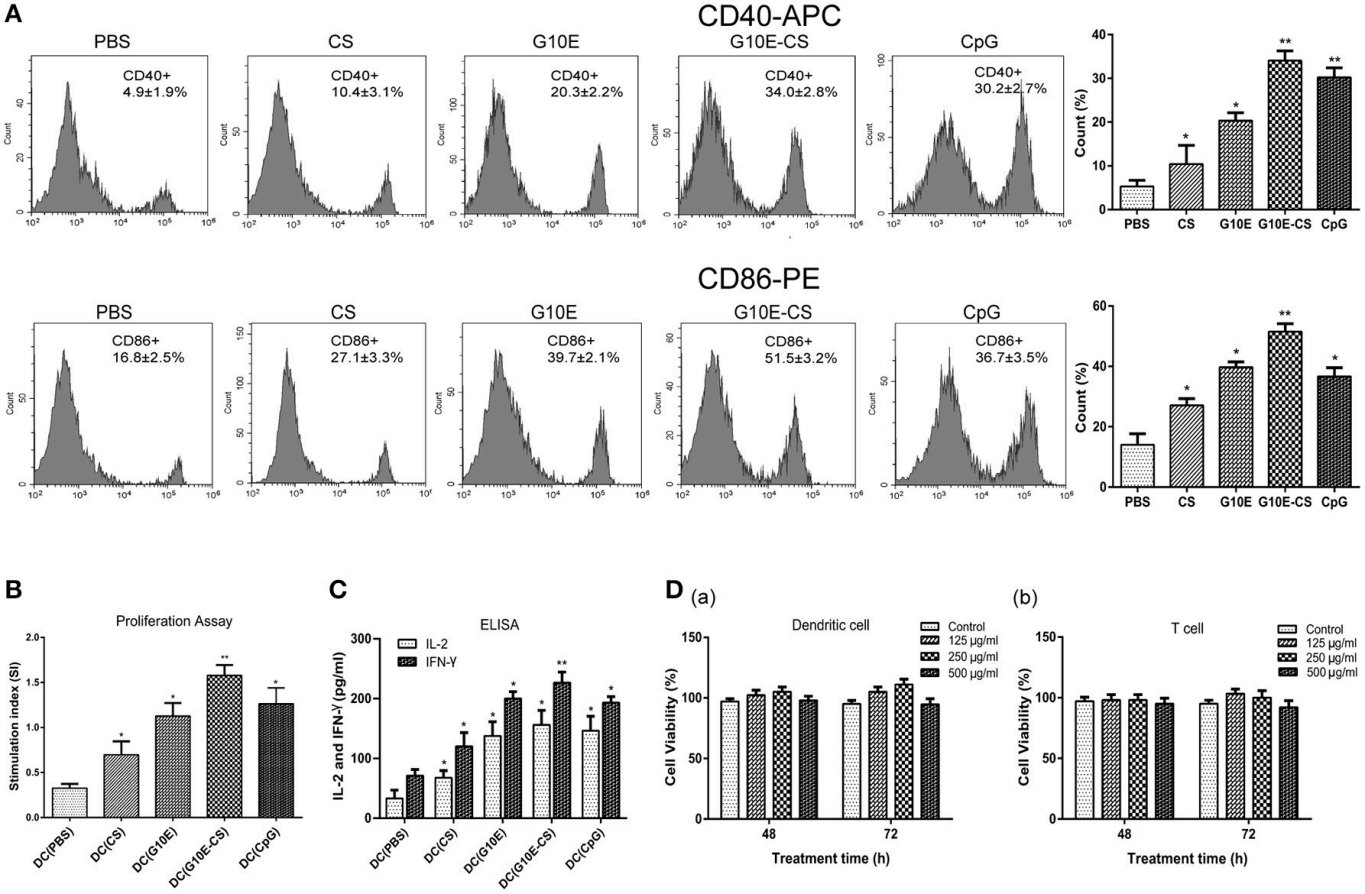
G10E-CS induce the activation of dendritic cells and lymphocyte *in vitro*. **(A)** The expression of the co-stimulatory molecules CD40 and CD86 on the surface of stimulated dendritic cells (DCs). DCs of C57BL/6 mice were incubated withG10E-CS, G10E, CS, CpG, or PBS for 48 h and the expression of CD40 and CD86 were detected by flow cytometry. Values represent mean ±SD (*n* = 5). *p < 0.05, **p < 0.01. **(B)** The analysis of T lymphocyte proliferation induced by stimulated DC cells. DCs of C57BL/6 mice were incubated with CpG, G10E-CS, CS, G10E, or PBS for 48 h and then they were cocultured with lymphocyte from C57BL/6 mice for 72 h. The lymphocyte proliferation was measured by CCK-8 assay. **(C)** The expression of cytokines (IFN-γ, IL-2) in the supernatant of lymphocytes induced by the stimulated DC cells. The supernatants of mixed cells were assayed for IL-2 and IFN-γ using ELISA assay. Data represent mean ±SD representative of three independent experiments. *p < 0.05, **p < 0.01. **(D)** Cytotoxicity of G10E-CS microparticles on dendritic cells (DCs) **(a)** and T cells **(b)**. DCs and T cells were incubated with different concentrations (125, 250, and 500 μg/ml) of G10E-CS microsphere for different incubation times (48 and 72 h) and cytotoxicity of G10E-CS was assessed by CCK-8 assay (mean ± SD, *n* = 5).

In order to determine if chitosan can induce dendritic cells (DC) activation *in vitro*, the expression of the co-stimulatory molecules CD40 and CD86 was detected by flow cytometry in stimulated DCs. Dramatic upregulation of CD40 and CD86 in DCs was observed after incubation with G10E-CS, G10E, CS, CpG (positive control) compared to PBS groups (*p* < 0.05; Figure 3A). G10E-CS microspheres achieved the highest level of the co-stimulatory molecules in dendritic cells (*p* < 0.01).

We further confirmed that CD86 and CD40 could provide activate lymphocytes. Hence, we assessed T cell proliferation and cytokines levels in supernatant of C57BL/6 mice lymphocytes after coculture with the above stimulated DC cells *in vitro*. As shown in Figure 3B, stimulated DCs provided more efficient co-stimulation of T cell proliferation, especially for G10E-CS stimulated cells (*p* < 0.01).

Supernatants of lymphocytes cocultured with the DCs showed that the CpG, G10E-CS, G10E microparticles could induce substantial production of IFN-γ and IL-2 compared to other groups (*p* < 0.05). The level of IFN-γ induced by G10E-CS microspheres (*p* < 0.01) was particularly noteworthy (Figure 3C).

Further, we studied the cytotoxic activity and biocompatibility of G10E-CS. Using CCK-8 assay, the viability of dendritic cells and T cells was analyzed by exposing them to different concentrations of G10E-CS (125, 250, and 500 μg/ml) for different incubation times (48 or 72 h). For DCs and lymphocytes, the results demonstrated that all the G10E-CS microspheres showed cell viabilities higher than 90% of the negative control (Figure 3D). Hence, G10E-CS microspheres were suggested to be safe for the mouse vaccine.

### G10E-CS induced robust *T. gondii* specific humoral immune responses

**Figure 4:**
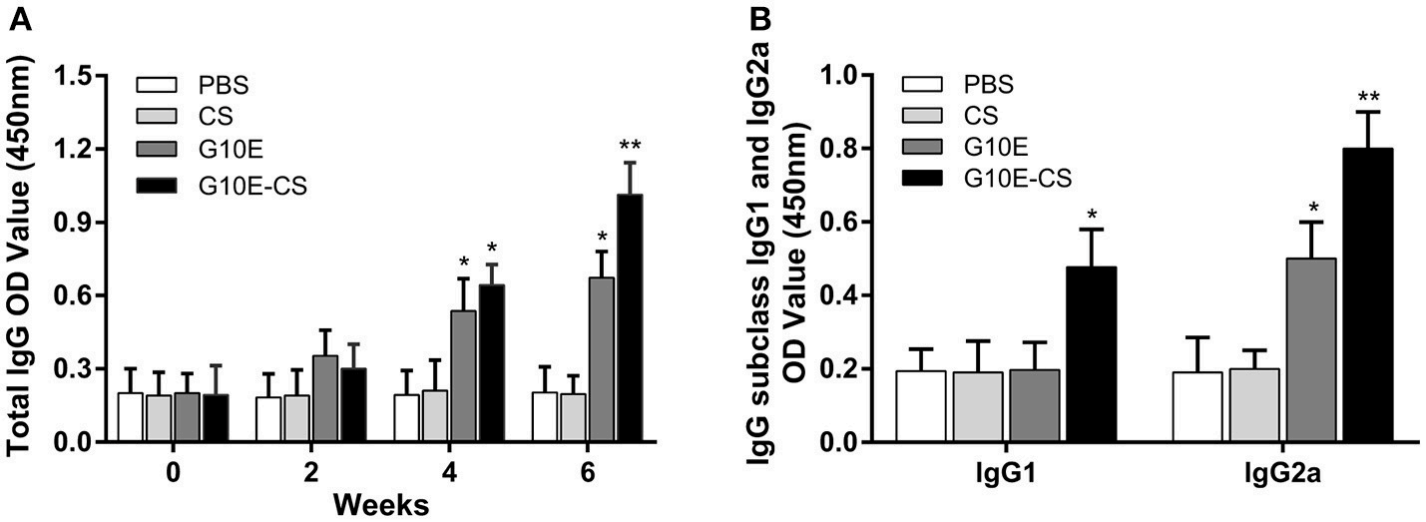
Humoral immune response induced in BALB/C mice after vaccination with G10E-CS. **(A)** Detection of total anti-GRA10 IgG antibody in the sera of immunized BALB/C mice. The mice were immunized intramuscular injection three times at 2 weeks interval with G10E, CS, G10E-CS, or PBS. Sera were collected from mice by retro-orbital bleeding at 0, 2, 4, and 6 weeks after immunization. The levels of *T. gondii*-specific IgG antibody in the sera of samples was used to determine by ELISA assay. **(B)** Detection of IgG subclass IgG1 and IgG2a antibodies in the sera of the immunized mice. Sera were collected at 2 weeks after the last vaccination. The level of IgG1 and IgG2a antibodies were measured by ELISA. Results are expressed as the mean of OD450 ± SD (*n* = 15) and are representative of at least three independent experiments. *p < 0.05, **p < 0.01.

Specific IgG in the sera of mice vaccinated intramuscularly with G10E-CS, G10E, CS, was measured every 2 weeks. A dramatically higher level of total IgG was observed in the sera of mice immunized with G10E-CS and G10E after the third immunization, in contrast to the other groups. G10E-CS immunized mice had the highest level of *T. gondii* specific IgG (*p* < 0.05) (Figure 4A).

Furthermore, the level of specific IgG1 and IgG2a sub-classes against G10E peptides was measured. As shown in Figure 4B, significant production of IgG2a and IgG1 was observed in the sera of mice immunized with G10E-CS (*p* < 0.01) compared with G10E group. These results indicated that mice immunized with G10E-CS microspheres generated Th1 polarize immune response with more IgG2a than IgG1.

### G10E-CS induced robust *T. gondii* specific cellular immune response

**Figure 5:**
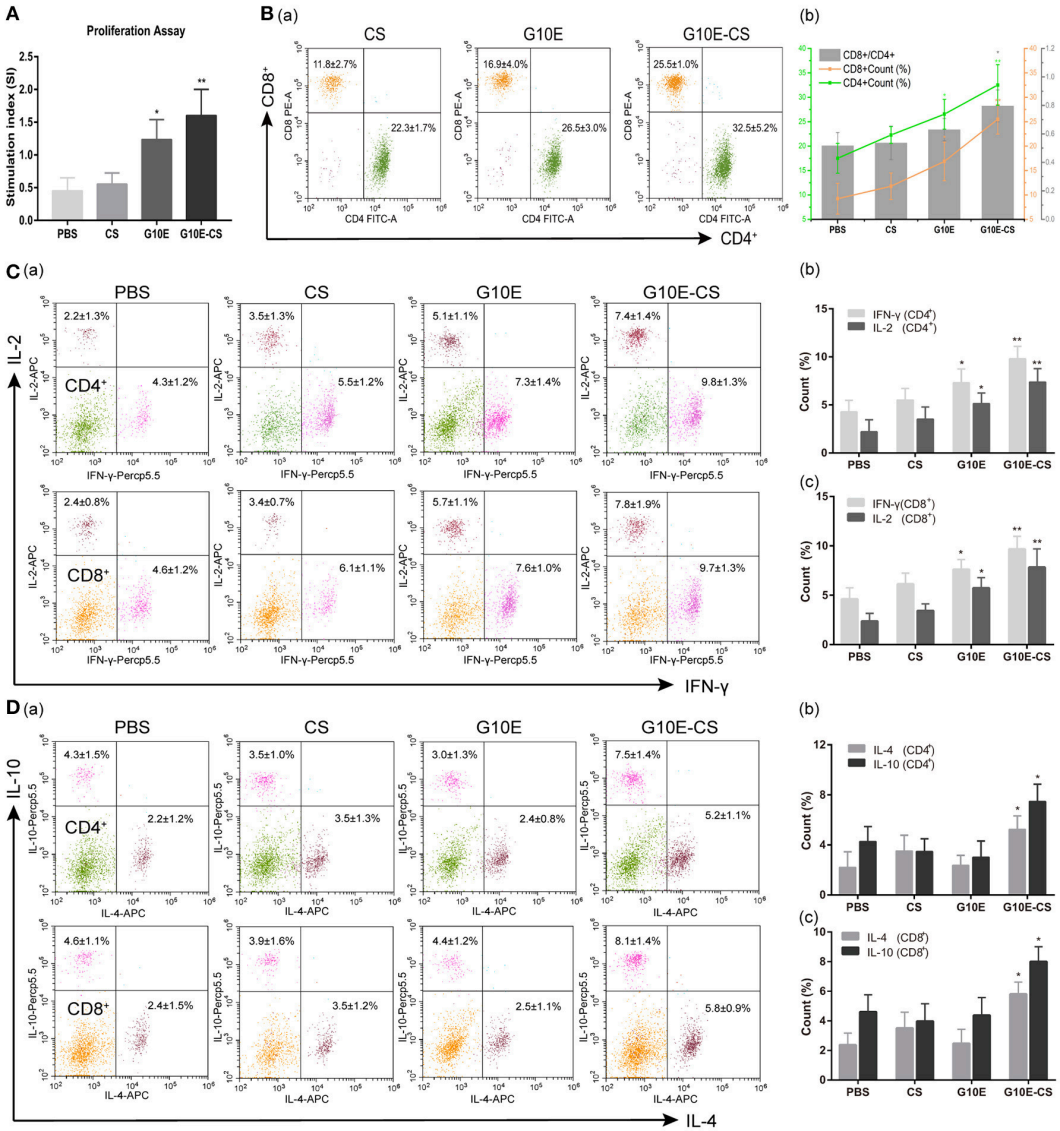
Cellular immune response induced in BALB/C mice after vaccination with G10E-CS. BALB/c mice were immunized with G10E-CS, CS, G10E, or PBS three times at 2-week intervals. Then splenocytes were harvested from three mice per group 2 weeks after the final immunization. Cellular immune responses were analyzed after stimulation with G10E peptides (10 μg/ml) for 72 h. **(A)** The lymphocyte proliferative response in immunized mice was measured by CCK-8 assay. The absorbance was detected at 450 nm and the stimulation index (SI) was calculated. **(B)** The percentages of T lymphocyte subsets **(a)** and ratio of CD8^+^/CD4^+^ **(b)** in immunized mice. The G10E stimulated splenocytes were stained with anti-mouse CD3-APC, anti- mouse CD4-FITC and anti-mouse CD8-PE for 30 min and cell population was analyzed by flow cytometry. **(C,D)** The production of cytokines (IFN-γ, IL-2) **(C)** and cytokines (IL-4, IL-10) **(D)** in the immunized mice. The splenocytes were stimulated with G10E peptides for 72 h in the presence of Cell Stimulation Cocktail to inhibit the secretion of cytokine into the extracellular and were fixed using an Intracellular Fixation & Permeabilization Buffer Set Kit. After stained with anti-mouse CD4-FITC, anti-mouse CD8-PE, anti-mouse IL-2 (APC), anti-mouse IFN-γ (PerCP-Cyanine 5.5), anti-mouse IL-4 (APC) and anti-mouse IL-10 (PerCP-Cyanine5.5) for 30 min, the cells population was analyzed by flow cytometry. Data represent the mean ± SD and splenocytes from three mice in each group were tested individually. *p < 0.05, **p < 0.01.

Antigen-specific proliferative responses of lymphocytes from immunized mice were detected by CCK-8 2 weeks after the third immunization and represented by the SI value as illustrated in Figure 5A. The level of lymphocyte proliferation in the mice immunized with G10E-CS (*p* < 0.01) and G10E (*p* < 0.05) was significantly higher than those of other groups. This result indicated that the T lymphocytes proliferation of mice vaccinated with G10E-CS was successfully stimulated.

To determine CD8^+^ or CD4^+^ T cells numbers after vaccination, the percentages of CD4^+^ and CD8^+^ T lymphocytes subsets in the spleen of immunized mice were evaluated by flow cytometry analysis. As shown in Figures 5Ba,b. the percentages of CD8^+^ and CD4^+^ T cells were both increased in mice immunized with G10E-CS and G10E compared to other groups (*p* < 0.05). G10E-CS-immunized mice also had the highest ratio of CD8^+^/CD4^+^ (*p* < 0.05). G10E-CS microspheres, therefore, could elicit more activation of CD8^+^ and CD4^+^ T cells in immunized mice than other vaccine formulations.

The production of cytokines in splenocytes supernatants of immunized mice was evaluated by Flow Cytometry. Significantly higher levels of IFN-γ and IL-2 were secreted by lymphocytes of the G10E-CS (*p* < 0.01) and G10E (*p* < 0.05) immunized mice compared to that of CS and PBS (Figures 5Ca,b). Interestingly, spleen cells from mice immunized with G10E-CS produced more IL-10 and IL-4 compared to other groups (*p* < 0.05; Figures 5Da,b).

### G10E-CS immunization against acute and chronic toxoplasmosis

**Figure 6:**
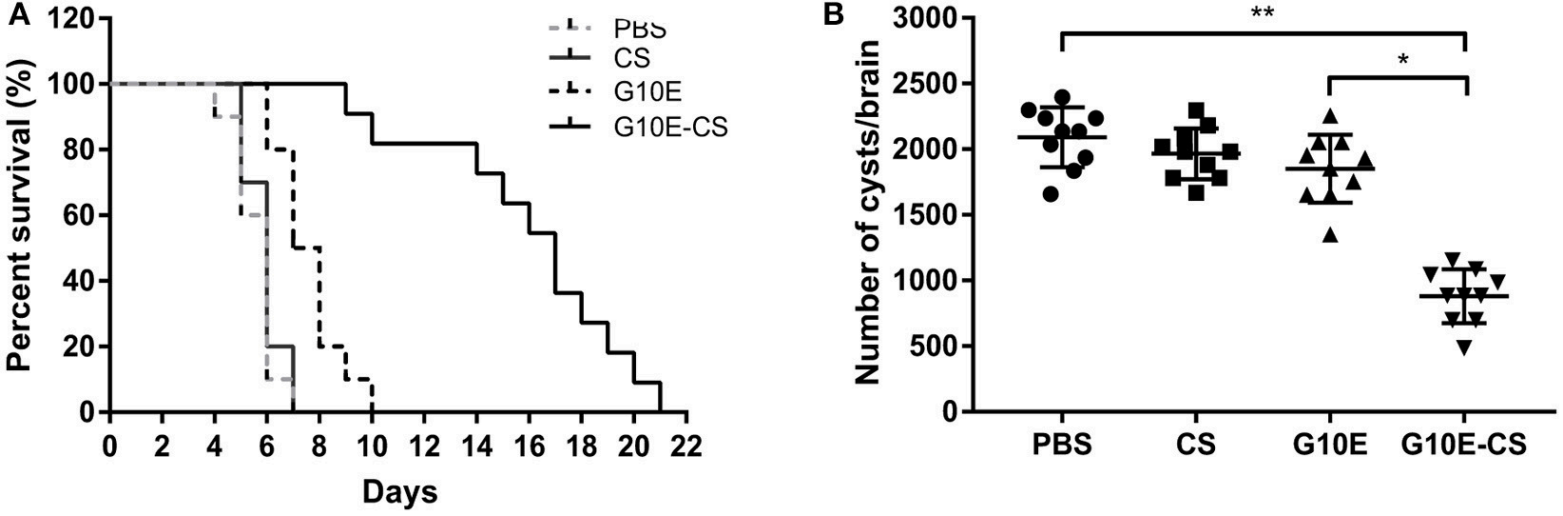
Protection of G10E-CS immunized mice against acute and chronic *Toxoplasma gondii* infection. **(A)** Survival rate of vaccinated mice after lethal RH strain tachyzoites challenge. Two weeks after the final immunization, 10 mice per group were intraperitoneally infected with 1 × 10^3^ tachyzoites of RH strain and observed daily for mortality. **(B)** The cyst number in the brain of mice after sublethal PRU strain cyst challenge. 10 mice per group were challenged orally with a sublethal dose of cysts (30 cysts) of the PRU strain (type II). Cyst load was counted from whole brain homogenates of mice 45 days after challenge. Data are representative results of three independent experiments and are represented as the means ± SD. *p < 0.05, **p < 0.01.

In order to evaluate if G10E-CS could confer effective protection against *T. gondii* acute and chronic infection, immunized mice were challenged with RH tachyzoites, 10 mice per group were challenge with 1 × 10^3^ RH tachyzoites 2 weeks after the last immunization. The survival curves of immunized mice challenged with 1 × 10^3^ RH tachyzoites 2 weeks after the last immunization were illustrated in Figure 6A. All mice vaccinated with PBS, G10E, or CS succumbed within 10 days of infection. In contrast, a significantly prolonged survival time (21 days) was observed in mice vaccinated with G10E-CS microspheres compared to other groups (*p* < 0.01). While only 3 days survival extension when mice immunized with only G10E without CS (10 days) compared to control group (*p* > 0.05).

The efficacy of this vaccine formulation in the context of chronic infection with *T. gondii* was also evaluated by challenging the immunized mice with 30 cysts of the PRU strain orally. On day 45 post challenge, as shown in Figure 6B, G10E-CS immunized mice had fewer cysts (882 ± 194 cysts) in brain than G10E (1852 ± 245 cysts), CS (1966 ± 183 cysts) and PBS (2091 ± 215 cysts) treated mice (*p* < 0.01). This result suggests that the G10-CS microsphere vaccine could significantly reduce the cyst form in mice chronically infected with *T. gondii*.

## Discussion

In this study, we first screened and constructed a MAP encoding three dominant B, CD8^+^ and CD4^+^ cell epitopes from *T. gondii* GRA10 (G10E). Chitosan microspheres were loaded with adequate G10E in an effort to protect the peptides from enzymatic degradation and release the G10E peptides stably. This complex was referred to as G10E-CS. *In vitro*, G10E-CS microspheres induced DC maturation and T cell activation with enhanced expression of costimulatory molecules (CD40 and CD86). *In vivo*, significantly prolonged survival and decreased brain cyst burdern were observed in mice vaccinated with G10E-CS, accompanied by strong humoral and cellular responses.

To control outbreaks of toxoplasmosis, caused by this intracellular protozoan parasite with its complex life cycle, the MAP vaccine, containing multiple epitopes, has many advantages over traditional vaccines (Henriquez et al., 2010; Skwarczynski and Toth, 2016). Currently, individual B (Darcy et al., 1992), CD4^+^ T (Grover et al., 2012), CD8^+^ T (Blanchard et al., 2008) cell epitopes have been able to induce partially protective immune responses against toxoplasmosis, and MAP vaccines are more likely to confer more robust protection (Bastos et al., 2016). GRA proteins of *T. gondii*, major components of both tachyzoites and bradyzoites, are related to host-parasite interaction and immune response. Dense granule protein 10 (GRA10) is essential for parasite growth with potential immunogenic capability, and it had not been studied as a candidate vaccine antigen prior to the work presented herein. In this study, we used bioinformatics and immunological methods to select nine B cell or T cell mimotopes of GRA10. Among them, three strongest immunogenic B and T cell epitopes, namely B cell epitope (TQSPPESRKKRRRSGKKKRGKRSV), CD8^+^ T cell epitope (LGYCALLPL), and CD4^+^ T cell epitope(SGFSLSSGSGVSVVE), were selected to construct the MAP, G10E. Peptide-based vaccines have some weaknesses. For example, they are poor immunogens, in and of themselves, and very susceptible to enzymatic degradation. Therefore, they need the assistance of an effective delivery system (Salvador et al., 2011; Skwarczynski and Toth, 2016).

Recently, a variety of delivery systems have been used for the formulation of peptide vaccines against *T. gondii*. Of particular interest, usage of nano- and micro-particles for vaccine delivery has rapidly become popular (Skwarczynski and Toth, 2016). Poly(lactide-co-glycolide) (PLG) has been tested as safe delivery systems to encapsulate recombinant SAG1 protein (Chuang et al., 2013) or rhoptry proteins (ROP18 and ROP38) (Xu et al., 2015) as controlled-release microparticle vaccines. Nanosized carriers have also been used to deliver parasite antigens to immune competent cells. Assembled GRA7-derived HLA-B^*^0702 -restricted epitopes into nanospheres (SAPN) (El Bissati et al., 2014) or loaded *T. gondii* extracts protein in porous nanoparticles (DGNP) (Dimier-Poisson et al., 2015) have been developed. All these delivery systems could protect protein epitopes against enzymatic degradation, and microspheres can sustain the stable release of antigen. Chitosan has particular advantages because easily assumes a spherical conformation, and is positively charged. It has been widely studied for vaccine delivery systems to increase the response to immunization with protein antigens, DNA plasmids, and bacterial derived toxins (Arca et al., 2009; Chua et al., 2012). There is comparatively little assessment of the utility of chitosan-based microparticles as vaccine carriers for peptides.

In our study, G10E-CS microspheres prepared by emulsion cross-linking were spherical in shape and had uniform diameter around 1,283nm. Size and zeta analysis of G10E-CS formulations confirmed that G10E was completely loaded into the core of the CS microspheres, and not simply adherent to their surfaces. These properties ensured no obvious degradation of G10E peptides during loading and release processes. This was further supported by PAGE analysis of released G10E. As a cationic polymer, chitosan can bind strongly to cell surfaces with a predominantly negative charge, thereby increasing residence time and releasing peptides in a sustained fashion in targeted cells (Koppolu et al., 2014). In this study, we observed a slow-release effect of G10E-CS microspheres. It is worth noting that nano- or micro-particles used in vaccines are often limited by their toxicity and the difficulty with which they are manufactured (Zhang et al., 2015a). These effects have been observed with chitosan microspheres in the past. Glutaraldehyde, the traditional cross-linking agent used in the production process of CS microparticles may be built into the vaccine carrier and show cell cytotoxicity (Agnihotri et al., 2004). To overcome this problem, we replaced glutaraldehyde with genipin, an aglucone of geniposide extracted from gardenia fruits and used in traditional Chinese medicine with demonstrated low cell cytotoxicity (Lin et al., 2013). Moreover, its low crosslinking speed with chitosan could avoid the occurrence of agglomeration (Jin et al., 2004). This difference led to the more uniform particle size observed herein. As expected, all concentrations of G10E-CS microspheres studied showed almost no cytotoxicity, a direct result of the choices of molecules with studies supporting good biocompatibility and low cytotoxicity.

Generally, immune responses mediated by CD4 ^+^ Th1 cell, especially the cytotoxic T lymphocyte (CTL) response, can directly suppress the growth of dividing tachyzoites and reactivation of the cysts of *T. gondii* (Ching et al., 2016). Thus, activation of T cells and production of Th1 cytokines are pivotal for the control of the parasite (Wang J. L. et al., 2017). Given the effective delivery system of the chitosan (CS) microspheres, the MAP (G10E) were stably released, inducing a strong cell-mediated immune response. In this study, we observed that T lymphocyte proliferation in mice immunized with G10E-CS microspheres was significantly higher than other groups. Meanwhile, percentages of CD8^+^ and CD4^+^ T lymphocyte were both increased dramatically in this group, together with the highest ratio of CD8^+^/CD4^+^ T cells. The ability of G10E-CS microspheres to stimulate T lymphocytes proliferation and differentiation was similar to that noted in a previous study (Xu et al., 2015), which encapsulated *T. gondii* ROP38/18 into PLG microparticles. The CD8^+^ T lymphocyte subset is important in responding to *T. gondii* infection and the helper CD4^+^ T subgroup appears to cooperate in the control of chronic infection by producing IFN- γ and redundant CD8^+^ T cells (Caetano et al., 2006).

IFN-γ plays a major role in protecting the infected host against *T. gondii* infection. Its secretion is directly proportional to the mortality rate of infected mice, because it can inhibit tachyzoite propagation during the early stages of infection by activating phagocytes such as macrophages, provide the appropriate cytokine environment (Innes et al., 2009; Sonaimuthu et al., 2016). IL-2 is essential for the development of cytotoxic CD8^+^ lymphocytes (Sa et al., 2013). As expected, we found that mice injected with G10E-CS generate the highest levels of IFN-γ and IL-2 in all immunized mice. Thus, we speculate that G10E-CS induces a stronger cell-mediated immune response than G10E due to enhanced delivery via the CS delivery system, and the response is stronger than CS alone due to the immunogenicity of the MAP, G10E. IFN-γ and IL-2 are critical for coordinating protective immunity against *T. gondii* (Luo et al., 2017). Many nano- and micro-particle vaccine formulations against *T. gondii* have observed similar results (Chuang and Yang, 2014; El Bissati et al., 2014; Dimier-Poisson et al., 2015; Xu et al., 2015).

Previous studies have also indicated a key role for vesicle size in determining the Th1/Th2 bias of the resulting immune response. Vaccination using influenza A antigen entrapped in the larger bilosome formulation (980 nm) resulted in a more biased Th1 response as measured by antigenspecific IgG2a serum levels and splenocyte IFN-γ production than vaccination using the smaller bilosomes (250 nm) (Mann et al., 2009). In this study, the size of G10E-CS microparticles was about 1,000 nm which result in Th1 bias immune response.

Th2-related IL-4 and IL-10 possess the antagonistic effect to IFN-γ (Bessieres et al., 1997), making this observation compelling. Interestingly, we also found spleen cells from mice immunized with G10E-CS produced slightly more IL-4, IL-10 than other groups. It has found that the early mortality of infected mice in the acute phase of toxoplasmosis is caused by severe inflammatory effects and lethal immunopathology provoked by IFN-γ, rather than parasitic infection itself (Ching et al., 2016). Even it has been recognized that a protective immune response against *T. gondii* infection requires a Th1 predominance response (Kumar et al., 1998; Meira et al., 2014). Th2-related cytokines play a vital role in downregulating this short-term deleterious inflammatory effect and reduce fatality following *T. gondii* infection (Neyer et al., 1997; Pinzan et al., 2015). Our findings are consistent with this conclusion. It is worth mentioning that GRA10 is found to translocate into the host cell nucleus and interact with signal transducers and promote activation of transcription factor 6 (STAT6). STAT6 plays a central role in the activation of the IL-4 response, triggering the expression of anti-apoptotic factors. In sum, mice immunized with G10E-CS have induced a combined Th1/Th2 immune response, skewed predominantly toward a Th1 response.

The critical role of antibody in immunity against *T. gondii* has long been recognized (Correa et al., 2007). Antigen-specific antibody can inhibit the tachyzoite directly and impair attachment to host cells (Sayles et al., 2000). In the present study, the increased production of total IgG, IgG2a and IgG1 were all observed in the sera of mice immunized with G10E-CS microspheres. As IgG1 is Th2 related and IgG2a is associated with Th1-driven immunity (Germann et al., 1995; Wang S. et al., 2017), our data supports the idea that G10E-CS microspheres generated Th1 polarize immune response with more IgG2a than IgG1.

Although there are some effective nano- or micro-particles vaccines against *T. gondii*, almost no delivery system can simultaneously act as a good adjuvant. A potent adjuvant required for *T. gondii* vaccines is able to elicit a Th1-type immune response (Verma and Khanna, 2013). It is exciting to find that chitosan can also serve as an attractive candidate adjuvant to induce dendritic cells (DCs) activation and, thereby, promote Th1 cellular immune responses (Riteau and Sher, 2016). In particular, internalized chitosan induces mitochondrial stress and increase reactive oxygen species (ROS) production, which act as a trigger for cGAS-STING pathway (Carroll et al., 2016). This pathway could mediate the production of IFN-γ, responsible for triggering the activation of DCs and T helper type 1 (Th1) immunity responses (Dubensky et al., 2013). DC activation is characterized by elevated surface expression of costimulatory molecules CD40 and CD86 (Reis e Sousa, 2006). The costimulatory molecules also have a key role in regulating T cell proliferation, cytokine production and generation of CTL (Chen and Flies, 2013). In our study, we found that bone-marrow-derived DCs exposed to G10E-CS microspheres *in vitro* showed drastically increased expression of CD40 and CD86. G10E-CS stimulated DCs could both induce efficient costimulation of T cell proliferation and substantial production of IFN-γ and IL-2. Therefore, G10E-CS microspheres could induce DC maturation and T cell activation *in vitro* by upregulating the expression of costimulatory molecules.

The survival rate of vaccinated mice against *T. gondii* challenge is considered the most direct way to assess a candidate vaccine (Zheng et al., 2017). In the present study, we found that mice vaccinated with G10E-CS microspheres have significantly prolonged survival time (21 days) against lethal challenge with the virulent RH strain of *T. gondii*. In contrast, mice immunized with PBS, G10E, or CS had all succumbed within 10 days after infection. Evaluation of the ability of G10E-CS to protect against chronic infection was also of interest. After challenging with a sublethal dose of cysts of the PRU strain, cyst burden in mice vaccinated with G10E-CS was greatly decreased (57.8% reduction) compared to the other groups. These results, taken together, proved that our G10E-CS microsphere vaccine was able to protect against both acute and chronic *T. gondii* infection.

In summary, in this study, G10E-CS microspheres have triggered a strong, mixed Th1/Th2 cellular and humoral immune response in immunized mice. At the same time, CS as a potent adjuvant, has also enhanced cellular immunity by eliciting Th1-type immune responses. Therefore, the humoral and cellular immune responses induced by G10E-CS microspheres were interrelated and synergistic in strengthening protection against *T. gondii*. Our results proved that chitosan microparticles loaded with MAPs could be a promising approach to vaccine development against *T. gondii*.

A limitation of this study was that the loading capacity (LC%) of the chitosan microsphere was low (15.3%). This may be due to liquid paraffin on the surface of the microspheres, which was hard to clean completely, potentially affecting the peptides contact with the microspheres. Interestingly, this loading capacity was slightly higher than that observed with chitosan-based microspheres loaded with other protein (Li et al., 2017). It will be necessary to increase the loading capacity to improve the protection efficiency of G10E-CS vaccine. Therefore, other optimized published methods for manufacturing protein-loaded chitosan particles can be used in future research.

## Authors contributions

HC and HY designed experiments. HY, JG, TW, and XS performed experiments and analyzed data. JG and HY wrote the manuscript. HC and YL revised the manuscript. CZ, HZ, and SH provided advice for experiment. All authors read and approved the final manuscript.

### Conflict of interest statement

The authors declare that the research was conducted in the absence of any commercial or financial relationships that could be construed as a potential conflict of interest.

## Supplementary material

Figure S1 The release of G10E peptides was analyzed by PAGE **(A)** and SDS-PAGE **(B)**. G10E-CS microspheres were dispersed in PBS (pH 7.4) using a shaking air bath (37°C,100 rpm) for 15 d. After centrifuging (5,000 rpm, 20 min), the microspheres precipitation at the bottom of the tube was collected to be analyzed by non-denaturing gel electrophoresis (PAGE) and denaturing gel electrophoresis (SDS-PAGE).

Table S1 Size and zeta potential of G10E-CS, CS and G10E. Size and zeta potential of chitosan microspheres loaded with G10E peptides (G10E-CS), chitosan microspheres (CS) and free G10E peptides.
