# Supplementary figures and images for: Chitosan Microsphere Used as an Effective System to Deliver a Linked Antigenic Peptides Vaccine Protect Mice Against Acute and Chronic Toxoplasmosis
Source: Front Cell Infect Microbiol. 2018 May 23;8:163. doi: 10.3389/fcimb.2018.00163 (PMC5974094; doi:10.3389/fcimb.2018.00163)

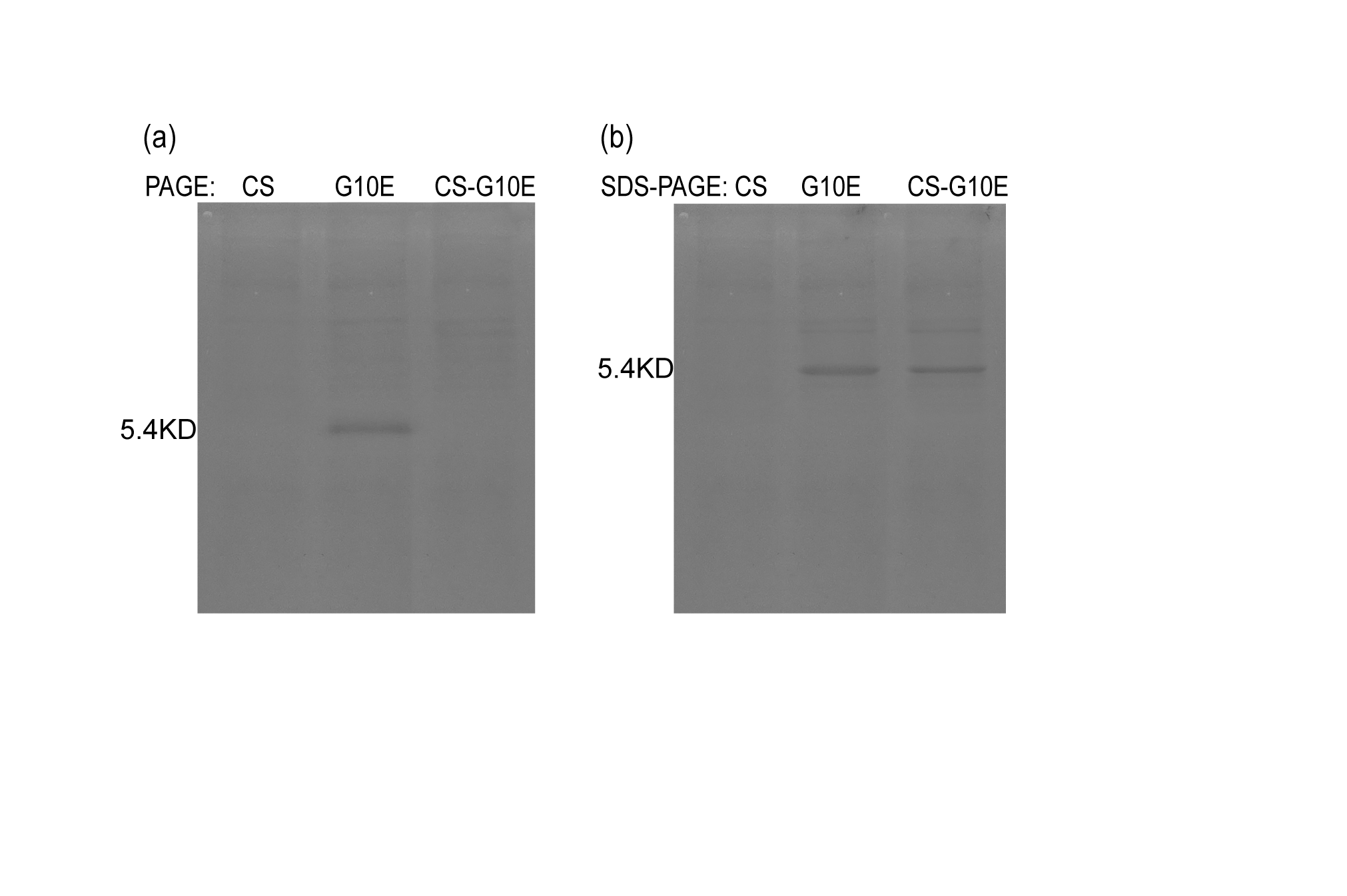

Supplement: Figure S1 — The release of G10E peptides was analyzed by PAGE (A) and SDS-PAGE (B). G10E-CS microspheres were dispersed in PBS (pH 7.4) using a shaking air bath (37°C,100 rpm) for 15 d. After centrifuging (5,000 rpm, 20 min), the microspheres precipitation at the bottom of the tube was collected to be analyzed by non-denaturing gel electrophoresis (PAGE) and denaturing gel electrophoresis (SDS-PAGE). [file Image_1.TIF]
