# Supplementary material for: Chitosan Microsphere Used as an Effective System to Deliver a Linked Antigenic Peptides Vaccine Protect Mice Against Acute and Chronic Toxoplasmosis
Source: Front Cell Infect Microbiol. 2018 May 23;8:163. doi: 10.3389/fcimb.2018.00163 (PMC5974094; doi:10.3389/fcimb.2018.00163)
Supplement: Table S1 — Size and zeta potential of G10E-CS, CS and G10E. Size and zeta potential of chitosan microspheres loaded with G10E peptides (G10E-CS), chitosan microspheres (CS) and free G10E peptides. [file Table_1.pdf]

TABLE S1丨Size and zeta potential of G10E-CS, CS and G10E[a].

	Size(nm)	Pdi[b]	Zeta potential(mV)
CS	1250 ± 280	0.169	+ 37.1 ± 5.2
G10E	0.41 ± 0.08	0.171	- 31.7 ± 9.1
CS-G10E	1283 ± 310	0.448	+ 36.0 ± 5.48

[a]Size and zeta potential of chitosan microspheres loaded with G10E peptides (G10E-CS), chitosan microspheres (CS) and G10E peptides. G10E-CS are chitosan microspheres prepared by emulsion cross-linking with G10E peptides in their core. The particle size and zeta potential of microspheres was determined in triplicate by Marvin particle size potentiometer.

[b]pdi: polydispersity index.

Data represent the mean ± SD.
