# Supplementary material for: Chitosan Microsphere Used as an Effective System to Deliver a Linked Antigenic Peptides Vaccine Protect Mice Against Acute and Chronic Toxoplasmosis
Source: Front Cell Infect Microbiol. 2018 May 23;8:163. doi: 10.3389/fcimb.2018.00163 (PMC5974094; doi:10.3389/fcimb.2018.00163)

**Table s1｜Size and zeta potential of G10E-CS, CS and G10E^a^**.

|  | **Size(nm)** | **Pdi^b^** | **Zeta potential(mV)** |
| --- | --- | --- | --- |
| CS | 1250±280 | 0.169 | +37.1±5.2 |
| G10E | 0.41±0.08 | 0.171 | -31.7±9.1 |
| CS-G10E | 1283±310 | 0.448 | +36.3±5.5 |

^a^Size and zeta potential of chitosan microspheres loaded with G10E peptides (G10E-CS), chitosan microspheres (CS) and G10E peptides. G10E-CS are chitosan microspheres prepared by emulsion cross-linking with G10E peptides in their core. The particle size and zeta potential of microspheres was determined in triplicate by Marvin particle size potentiometer.

^b^pdi: polydispersity index.

Data represent the mean ± SD.

参考：


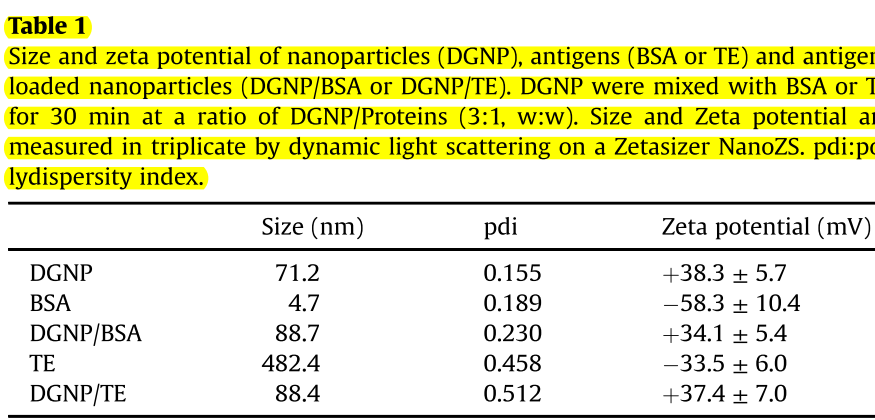

Supplement: Supplementary file 3 [file Table_2.DOC]
